# Clinical importance of serum miRNA levels in breast cancer patients

**DOI:** 10.1007/s12672-024-00871-y

**Published:** 2024-01-27

**Authors:** Fatih Turkoglu, Akin Calisir, Bahadir Ozturk

**Affiliations:** 1 Department of General Surgery, Faculty of Medicine, Selcuk University, Akademi Mahallesi Yeni İstanbul Caddesi No:313, Selçuk Üniversitesi Alaeddin Keykubat Yerleşkesi, Selçuklu, Konya, 42130 Turkey; 2 Department of Biochemistry, Faculty of Medicine, Selcuk University, Konya, Turkey

**Keywords:** Breast cancer, Microrna, Prognosis, Biomarker

## Abstract

There is limited data on the relationship of miRNAs with parameters that may affect surgical management or reflect tumour prognosis. It was aimed to evaluate serum miRNA levels in breast carcinoma cases and reveal the relationship between these levels and prognosis-related factors such as the histological type of the tumour, estrogen receptor, progesterone receptor, Ki-67 index, HER-2neu, E-cadherin, tumour size, CK5/6, CA15.3 levels, number of tumour foci, number of metastatic lymph nodes, and status of receiving neoadjuvant therapy. Thirty-five patients with a histopathologically confirmed breast carcinoma diagnosis in the case group and 35 healthy individuals in the control group were examined. miR-206, miR-17-5p, miR-125a, miR-125b, miR-200a, Let-7a, miR-34a, miR-31, miR-21, miR-155, miR-10b, miR-373, miR-520c, miR-210, miR-145, miR-139-5p, miR-195, miR-99a, miR-497 and miR-205 expression levels in the serum of participants were determined using the Polymerase Chain Reaction method. While serum miR-125b and Let-7a expression levels were significantly higher in breast cancer patients, miR-17-5p, miR-125a, miR-200a, miR-34a, miR-21, miR-99a and miR-497 levels were significantly lower in them. The Let-7a expression level had a statistically significant relationship with breast cancer histological type and HER-2neu parameters, miR-17-5p, miR-125b, Let-7a, miR-34a, miR-21 and miR-99a levels with E-cadherin, miR-34a, miR-99a and miR-497 with CA15.3, miR-125b, miR-200a and miR-34a with the number of metastatic lymph nodes, miR-125a with the number of tumour foci and miR-200a with the status of having the neoadjuvant therapy. Serum miR-17-5p, miR-125a, miR-125b, miR-200a, Let-7a, miR-34a, miR-21, miR-99a and miR-497 expression levels were determined to have predictive and prognostic importance in breast cancer.

## Introduction

 Breast cancer is the most commonly diagnosed type of cancer, accounting for 23% of all cancer cases diagnosed in women worldwide, and is the most common cause of cancer-related deaths [1–3]. Invasive ductal carcinoma (IDC) is the most common type of breast cancer [4].

There have been many clinical and radiological advances made in the early detection of breast cancer. There are many diagnostic methods such as mammography, magnetic resonance imaging, ultrasound, computed tomography, positron emission tomography and biopsy. However, despite advances, these techniques have some limitations, such as being expensive, time-consuming, and some of them not being suitable for young women [5]. This situation has encouraged researchers to look for different methods of diagnosis and treatment of diseases today, and the studies in this field have deepened as molecular biological techniques have developed. Molecular target-based studies have focused on advances in microRNA (miRNA) expression profiling because of their important role in tumour development and metastasis [6]. miRNAs are defined as single-stranded, short ncRNAs (non-codingRNAs) of 19–25 nucleotide in length, produced from endogenous transcripts, that regulate gene expression by stopping the translation of mRNAs (messenger RNAs) or by accelerating their degradation [7, 8]. The first microRNA (miRNA) was discovered in 1993 when two independent studies determined that the Caenorhabditis elegans heterochronic gene lin-4 is a small non-coding RNA (ncRNA) [9].

Many miRNA subtypes have been identified to date. Circulating levels of some of these subtypes have been determined to be significant in the diagnosis, treatment and prognosis of breast tumours. Breast cancer is a complex disease caused by the progressive accumulation of multiple gene mutations combined with epigenetic dysregulation of critical genes and protein pathways [10]. Among the investigated miRNAs in terms of breast cancer, while studies showed miR-21, miR-155, miR-10b, miR-373 and miR-210 with oncogenic functions, miR-206, miR-17-5p, miR-125a, miR-125b, miR-200a, Let-7a, miR-34a, miR-31, miR-520c, miR-145, miR-139-5p, miR- 195, miR-99a, miR-497 and miR-205 were shown to have tumour suppressor functions [11–32]. Recent studies have proven that changes in miRNA expression have a close relationship with various types of cancer [33–36]. Although there are studies evaluating miRNA levels in colorectal and gastric cancer in terms of histopathological characteristics, there is limited data on the relationship between miRNAs and parameters that may affect the treatment protocol or reflect tumour prognosis [37, 38]. Molecular therapy methods are an important development in cancer treatment. However, the functions of miRNAs are complex because they effectively regulate and control hundreds of genes, including oncogenes and tumour suppressor genes [39]. Furthermore, they might show oncogene, tumour suppressor gene or both oncogene and tumour suppressor gene behaviour in terms of cancer activity [40]. In our study, we aimed to evaluate serum miRNA levels in breast carcinoma cases and then reveal the relationship between these levels and histopathological factors that show prognosis, and to find out the role of miRNAs, whose serum levels have been determined, in cancer biogenesis.

## Materials and methods

After obtaining ethical approval from the medical ethics committee on 17.06.2020 with the decision number 2020/264 (Medical Faculty Ethics Committee of the concerned University), the study protocol was explained to all participants who agreed to participate in this study, and written informed consent was obtained from them. The study was carried out with a total of 70 women. Blood samples were collected from the patients who visited the Department of General Surgery, Faculty of Medicine of the concerned University between July 2020 and December 2021. Two groups were formed. While the first group consisted of 35 patients who underwent a modified radical mastectomy, whose histopathological diagnosis of breast carcinoma was confirmed, the second group consisted of 35 healthy individuals with no history of malignancy and additional disease. While patients with initial diagnosis (non-relapse) breast cancer undergoing a modified radical mastectomy procedure were included in the study, those who were younger than 18 years of age, those whose diagnosis of breast cancer was not histopathologically proven, those with relapse cases or those with missing data in their files were excluded from the study.

The study was carried out with the prospective method and the Declaration of Helsinki principles were followed during the study. Serum samples of the patients were used in our study. Information on the participants’ demographic characteristics, prognosis, and histological diagnoses was collected through face-to-face interviews with individuals and by examining the pathology and observation reports of the patient group. Participants were also examined in terms of demographic and social characteristics such as age, number of children, obesity, family history, status of physical activity, smoking and alcohol consumption. In the classification of obesity, the limit value for body mass index (BMI) was taken as 30 kg/m² [41]. In terms of physical activity, those who had a mean exercise time of 150 min a week were considered physically active [42, 43]. Blood samples were collected from the patient group in the preoperative period. The expression of hormone receptors was examined using the method of immunohistochemistry [44]. Estrogen and progesterone receptors were considered positive if 10% or more of the cell nucleus were stained at x10 magnification and the human epidermal growth factor receptor (HER-2neu) was considered positive if it was scored as + 3 [45]. Those with a Ki-67 index greater than 14% were considered positive [46]. Histological typing was performed using the approach of previously published research [47]. Those with a + 3 and + 4 score of E-cadherin were considered positive [48]. Those with a CA15.3 value of more than 30U/ml were considered high [49]. miR-206, miR-17-5p, miR-125a, miR-125b, miR-200a, Let-7a, miR-34a, miR-31, miR-21, miR-155, miR-10b, miR-373, miR-520c, miR-210, miR-145, miR-139-5p, miR-195, miR-99a, miR-497 and miR-205 expression levels in the serum of breast cancer patients and control group were determined using the PCR (polymerase chain reaction) method [50].

The method used in the previous study on the comparison of the microRNA spectrum between serum and plasma was used to obtain plasma [51, 52]. The obtained plasma samples were stored at − 80 °C until the day of the study. miRNA isolation was performed in accordance with the procedure using the RNeasy mini kit (Qiagen) as recommended in the manufacturer’s instructions [53, 54]. The purity and concentration of the isolated miRNAs were checked using a NanoDrop spectrophotometer (Quawell, Q-5000) and stored at − 80 °C until further evaluation. miScript II reverse transcription kit (Qiagen) was used to obtain reverse transcription and complementary DNA (cDNA). The cDNA concentration and purity obtained by following the kit’s usage protocols were determined using a NanoDrop spectrophotometer (Quawell, Q-5000) and stored at -20 °C until a quantitative real-time polymerase chain reaction (qPCR) was performed [55]. qPCR was performed using MiScript primer assay for miRNAs and MiScript SYBR Green PCR kit (Qiagen) for reaction. Also, RNU44 (SNORD44) was used as the endogenous control for the normalization of the expression levels of the examined miRNAs. The reaction for MiScript primers was carried out using cDNA in a concentration adjusted to 2 nanograms /millilitre and a total volume of 20 µl. The thermal reaction conditions were such that 15 min were at 95 °C, followed by 15 s at 94 °C for 40 cycles, 30 s at 55 °C and 34 s at 70 °C. Fluorescence was detected in the qPCR system (Light Cycler 96 system, Roche). Expression levels of investigated miRNAs were evaluated using the ∆Ct method [56]. The cycle of threshold (Ct) is the number of qPCR cycles required for the fluorescent signal to pass a certain threshold [57]. ∆Ct was calculated by subtracting the Ct values of SNORD44 from the Ct values of the studied miRNAs. Ct and ∆Ct values are cycle thresholds used to indicate miRNA expression levels.

### Statistical analysis

All data were analyzed by using the IBM SPSS Statistics v.25.0 program. Data were used to compare the patient and control groups via the software. Using the obtained Ct (Cycle of threshold) values, the ∆Ct values of the groups were calculated by formulating with the IBM SPSS Statistics v.25.0 program. The comparison of the groups was evaluated using the chi-square test. Pearson product-moment and Spearman-Brown rank correlations were calculated to determine the direction and the level of the relationships between independent variables. Furthermore, statistical significance values were calculated at the 95% confidence interval of the relations between the variables (*p* < 0.05 was accepted).

## Results

A total of 70 individuals participated in the study, and all of them were female. A cancer group consisting of 35 individuals newly diagnosed with breast cancer and a healthy control group consisting of 35 individuals without any additional disease were formed The social and demographic characteristics of the participants are presented in Table 1. There was no statistically significant difference between the participants in terms of social and demographic characteristics (*p* > 0.05).

**Table 1:** Demographic and social characteristics of the participants

Demographic and social characteristics		Patient		Control		P value
		N	%	N	%	
Age	≤ 40	6	17.1	24	68.5	0.061
	> 40	29	82.9	11	31.5	
Number of children	≤ 1	18	51.4	27	77.1	0.076
	> 1	17	48.6	8	22.9	
Smoking status	Yes	14	40	8	22.9	0.198
	No	21	60	27	77.1	
Status of alcohol consumption	Yes	10	28.5	3	8.6	0.065
	No	25	71.5	32	91.4	
Obesity	Obese	18	51.4	13	37.2	0.116
	Not obese	17	48.6	22	62.8	
Family history	Yes	9	25.8	5	14.3	0.370
	No	26	74.2	30	85.7	
Physical activity status	Active	16	45.7	20	57.1	0.274
	Not active	19	54.3	15	42.9	

In our study, the expression levels of miR-206, miR-497, miR-17-5p, miR-125a, miR-125b, miR-200a, Let-7a, miR-34a, miR-31, miR-21, miR-155, miR-10b, miR-373, miR-520c, miR-210, miR-145, miR-139-5p, miR-195, miR-99a, and miR-205 were measured in blood serum. When the data were evaluated, the serum expression levels of miR-125b and Let-7a, measured using the ∆Ct values of the microRNAs used in the study, were found to show a statistically significant increase in the patient group compared to the control group, whereas there was a statistically significant decrease in the expression levels of miR-17-5p, miR-125a, miR-200a, miR-34a, miR-21, miR-99a, and miR-497 (Table 2). No statistically significant difference was found in expression values of miR-206, miR-31, miR-155, miR-10b, miR-373, miR-520c, miR-210, miR-145, miR-139-5p, miR-195 and miR-205 (*p* > 0.05).

**Table 2:** Mean ∆Ct and p values of statistically significant miRNAs (*p* < 0.05)

M miRNA	Mean ∆Ct		P value
	Patient	Control	
miR-17-5p	4.738	5.167	**0.035**
miR-125a	1.824	2.049	**0.036**
miR-125b	5.673	4.313	**0.001**
miR-200a	7.790	8.484	**0.020**
Let-7a	6.305	4.963	**0.004**
miR-34a	12.532	12.705	**0.009**
miR-21	10.467	11.353	**0.001**
miR-99a	11.660	11.925	**0.005**
miR-497	8.238	8.814	**0.017**

Statistically significant p values are written in bold

A total of nine statistically significant miRNAs were detected with a 95% confidence interval (*p* < 0.05). These detected miRNAs were compared with the pathological and clinical parameters of breast cancer patients (Table 3). As the positivity of E-cadherin increased in the pathological diagnosis of the patients, miR-17-5p expression levels were found to be higher (*p* < 0.05). As the number of tumour foci increased, miR-125a levels were found to be lower in patients (*p* < 0.05). miR-125b levels were found to be higher in patients with negative E-cadherin values (*p* < 0.05). Similarly, miR-125b levels were high in patients with a high number of metastatic lymph nodes (*p* < 0.05). However, miR-200a levels were found to be higher in patients with a low number of metastatic lymph nodes (*p* < 0.05). Furthermore, patients who received neoadjuvant therapy had statistically significantly lower miR-200a levels than those who did not (*p* < 0.05). In the pathological diagnosis of the patients, an increase in the incidence of invasive ductal carcinoma was found to be associated with an increase in Let-7a (*p* < 0.05). Furthermore, Let-7a expression was higher in patients with HER-2neu amplification positivity and E-cadherin positivity in the pathological diagnosis (*p* < 0.05). As the positivity of E-cadherin increased in the pathological diagnosis of the patients, the miR-34a expression level was found to be higher (*p* < 0.05). miR-34a was high in patients with high CA15.3 levels and a high number of metastatic lymph nodes (*p* < 0.05). MiR-21 level was higher in patients who had E-cadherin positivity in their pathological diagnosis (*p* < 0.05). E-cadherin-positive patients compared to negative ones and those with CA15.3 levels in the normal range compared to those with higher levels were found to have higher levels of miR-99a(*p* < 0.05). miR-497 levels were also high in patients with high CA15.3 levels (*p* < 0.05). No significant relationship was found between the miRNAs whose expression levels were examined and estrogen receptor, progesterone receptor, ki-67 index, lesion size, and CK5/6 parameters (*p* > 0.05).

**Table 3:** Relationship between clinical and pathological characteristics of breast cancer patients and miRNA levels (*p* < 0.05)

miRNA factors		miR17-5p	miR125a	miR125b	miR200a	Let 7a	miR34a	miR21	miR99a	miR497
Histological type	Invasive ductal CA	24.33	23.02	26.68	30.55	27.24	34.44	32.21	33.09	29.58
	Non-invasive ductal CA	25.09	22.80	26.71	30.43	27.13	34.26	31.44	33.08	30.37
	p	0.473	0.327	0.056	0.399	0.003	0.245	0.575	0.332	0.870
	r	− 0.147	0.200	− 0.379	0.173	0.558	0.236	0.115	0.198	− 0.034
HER-2neu	Her-2neu ( +)	26.65	23.98	27.98	30.53	29.77	34.38	33.12	33.13	30.52
	Her-2neu (−)	27.49	23.29	29.09	30.60	28.19	34.93	35.98	34.33	31.16
	p	0.922	0.882	0.332	0.164	0.048	0.239	0.313	0.547	0.124
	r	− 0.020	0.031	− 0.198	− 0.281	0.363	− 0.239	− 0.206	− 0.124	− 0.309
E-cadherin	E-Cadherin ( +)	26.69	23.02	26.68	30.55	28.06	34.44	32.28	33.34	29.58
	E-Cadherin (−)	24.33	23.63	27.62	29.98	27.13	34.03	32.21	33.09	31.17
	p	0.034	0.095	0.003	0.381	0.027	0.019	0.047	0.006	0.620
	r	0.416	− 0.334	− 0.552	0.179	0.434	0.455	0.378	0.520	− 0.102
Ca15.3	High	27.08	23.51	25.80	30.34	26.71	34.64	32.60	33.03	30.18
	Normal	23.85	22.75	26.77	30.92	27.24	34.39	31.75	33.27	30.06
	p	0.478	0.202	0.115	0.080	0.244	0.042	0.168	0.013	0.018
	r	0.282	0.234	− 0.018	− 0.081	− 0.114	0.060	0.172	− 0.217	0.274
Number of met L.N	Multiple	26.68	24.09	27.84	30.26	28.62	35.02	32.82	33.46	30.93
	Single	25.93	23.10	26.66	30.55	27.82	34.06	31.54	33.02	30.41
	p	0.363	0.387	0.045	0.037	0.150	0.048	0.866	0.391	0.671
	r	0.186	0.177	0.381	− 0.396	0.291	0.364	0.035	0.175	0.087
Foci	Multisentric	24.54	22.93	26.51	30.27	26.82	34.39	32.48	32.92	29.21
	Solid	25.61	23.88	26.45	30.32	27.32	34.12	31.49	32.87	30.28
	p	0.422	0.006	0.144	0.157	0.204	0.101	0.208	0.435	0.467
	r	− 0.027	− 0.206	0.336	− 0.180	− 0.196	0.333	0.218	0.269	− 0.128
Status of receiving neoadjuvant CT/RT	Yes	26.90	23.84	27.78	29.09	28.67	33.28	34.35	32.62	29.62
	No	27.04	24.66	28.56	30.91	28.10	34.57	32.88	34.21	29.76
	p	0.317	0.902	0.209	0.024	0.765	0.597	0.162	0.570	0.840
	r	− 0.204	− 0.025	0.255	− 0.441	0.062	− 0.109	0.283	− 0.117	− 0.042

Statistically significant p values are written in bold

p: significance value; r: correlation coefficient; MET L.N.: metastatic lymph node; CA: carcinoma; CT/RT: chemotherapy/radiotherapy

## Discussion

This study indicated the potential of utilizing miRNAs as reliable biomarkers, offering both predictive and prognostic significance in the context of breast cancer.

Although there are surgical and non-surgical treatment methods used for breast cancer, there are difficulties with the early detection of breast tumours [58]. Detection at an early stage enables a better treatment outcome. miRNAs in different body fluids have been proven to have a significant relationship with the pathological characteristics of cancer [59–61].

In the existing literature, several studies suggest a dual role for miR-17-5p, exhibiting both tumour suppressor and oncogenic behaviours across various cancer types [62–65]. Our study’s findings reveal a significant decrease in miR-17-5p levels within the breast cancer group compared to the control group, indicating a downregulation akin to that of a tumour suppressor. Furthermore, patients with positive E-cadherin, which ensures the stability of the cell in its pathology, had higher miR-17-5p levels than those with negative E-cadherin. The high level of this miRNA type suggests that the cell is more stable in breast cancer patients, a decrease in cell mobility, separation and migration occurs. In their study, Wu et al. showed that inhibition of miR-17-5p led to an increase in the amount of E-cadherin and a regression in breast cancer progression [66]. There is a need for more studies to reveal the relationship between MiR-17-5p and E-cadherin.

MiR-125a is known for its tumour-suppressor properties [13, 67]. MiR-125a levels decreased in the patient group compared to the control group in our study. Hsieh et al. previously identified miR-125a as a prognostic biomarker with tumour suppressor properties in breast cancer [68]. In addition to their study, the decrease in the expression level of miR-125a, which was determined to have a statistically negative relationship with the number of breast cancer foci in our study, and the increase in the number of tumour foci were shown to be multicentric. In line with these data, given the potential higher likelihood of multicentricity in individuals with low miR-125a levels, it could be beneficial to take this into account during the planning of breast-conserving surgery. This consideration aims to mitigate local recurrence rates and ensure the thorough removal of tumours, minimizing the risk of residual lesions in patients.

Another type of miRNA, namely miR-125b, showed increased expression levels in the patient group compared to the control group, as determined in our study. miR-125b values were higher in patients with negative E-cadherin and those with a high number of metastatic lymph nodes. This suggests that the increase of miR-125b might increase tumour spread and the number of metastatic lymph nodes by negatively affecting cell stability. These data support each other in terms of the increase in the tumour’s ability to migrate. Although the irregular expression of miR-125b in various cancers has been proven in the literature, its mechanism of action is not yet fully clarified [69]. In some studies, miR-125b expression was found to be associated with breast cancer’s chemotherapeutic resistance [70]. According to our study findings, miR-125b emerges as a potential biomarker associated with a poor prognosis. High levels of miR-125b may signify a more aggressive tumour. Therefore, incorporating this information into the consideration of adjuvant therapy post-surgery could contribute to more informed and targeted treatment planning.

There was a decrease in the MiR-200a levels in the patient group compared to the control group. Although miR-200a is a miRNA known as a tumour suppressor, there was a negative relationship between the number of metastatic lymph nodes and the status of having the neoadjuvant chemotherapy/radiotherapy treatment in our study [71]. In other words, miR-200a expression levels were found to be lower in patients with a high number of metastatic lymph nodes and those who received neoadjuvant therapy, compared to patients with a low number of metastatic lymph nodes and those who did not receive neoadjuvant therapy. The fact that breast cancer patients had low levels of miR-200a and that low miR-200a levels were associated with multiple lymph node metastases in our study is in line with the literature on miR-200a, which has been shown to act as a tumour suppressor.

In our study, Let-7a levels increased in the patient group compared to the control group. Let-7a has been noted to suppress invasion and migration in breast cancer cells in the literature [72]. It has a statistically significant relationship with histological type, HER-2neu and E-cadherin. In this respect, Let-7a, together with miR-34a, was the miRNA variety that had a significant relationship with the most parameters. Its increased levels in circulation are associated with more invasive ductal carcinoma histological type, HER-2neu and E-cadherin positivity. Our findings revealed that Let-7a, which was suggested to act as a tumour suppressor in previous studies, is associated with increased HER-2/neu positivity levels, an indicator of poor prognosis, and increased levels of E-cadherin positivity, which is a good prognosis indicator [73, 74]. miR-34a, the other miRNA type whose expression level was examined, is known as a tumour suppressor [63, 75, 76]. According to the statistically significant data obtained in our study, the level of miR-34a decreased in the patient group. miR-34a expression was found to be high in patients with positive E-cadherin levels, a high number of metastatic lymph nodes and a high CA15.3 tumour marker in their pathology. This miRNA also gave statistically significant results with three parameters similar to Let-7a. Accordingly, miR-34a was a predictor of good prognosis in terms of E-cadherin positivity, and poor prognosis in terms of the number of metastatic lymph nodes and the level of CA15.3. The reason for this contradictory situation may be that the prognostic behaviour of breast cancer is multifactorial and has not yet been fully revealed. This suggests that these miRNAs may have much more complex functions that need to be explained and investigated.

miR-21 levels decreased in the patient group compared to the control group in our study. It was highly expressed in patients with positive E-cadherin compared to those with negative E-cadherin. This suggests that miR-21 has a role in providing cell stabilization. Given that existing studies propose miR-21’s role as an oncogene, and our study did not align with this observation, it is believed that there is a need for further studies on miR-21 [26, 77, 78].

Several studies in the literature affirm the tumour-suppressor role of miR-99a [22, 79, 80]. MiR-99a levels decreased in the patient group compared to the control group in our study. This shows that there is a similarity between our study and the literature. Moreover, miR-99a levels were found to be high in patients with positive E-cadherin and normal CA15.3 levels. Thus, the findings supported the idea that miR-99a is a good prognostic factor with its protective effect on the tumour and preventive effect on the spread.

Levels of miR-497, shown to decrease in the patient group compared to the control group, were more expressed in patients with higher CA15.3 levels compared to those with normal levels. It represented a poor prognosis due to its high levels in patients with high CA15.3 levels in our study. In the literature, a reduced expression level has been reported to be consistent with malignancy [81]. The observed contrast with CA15.3 levels suggests that further clarification of this phenomenon may be achieved through an increased number of studies.

Given all these data, it has been revealed that much more research and development on miRNAs can play an important role in the treatment and prognosis of breast cancer, as in other cancer types. Today, remarkable success has been achieved in revealing the human genome. Research on genetics continues to increase. This situation, by controlling miRNA expression levels, contributes to the development of new treatment programs and to predetermine the population that may be diagnosed with breast cancer. Moreover, enhanced predictability is anticipated to alleviate the financial burden associated with combating breast cancer, a prevalent form of cancer among women.

The limitations of our study were the relatively small number of participants, the lack of long-term follow-up and the presence of no ethnic diversity among the participants. We think that conducting studies with bigger sample numbers or on different races might provide more inclusive data.

## Conclusion

In conclusion, expression levels of serum miR-17-5p, miR-125a, miR-125b, miR-200a, Let-7a, mir-34a, miR-21, miR-99a and miR-497 determined using real-time PCR method have been shown to be biomarkers with predictive and prognostic importance in terms of the relationship between biochemical and histopathological parameters in breast cancer. Even if no lymph node is detected in preoperative radiological imaging in patients with high miR-125b and miR-34a levels and low miR-200a levels, dissection should be carefully evaluated, taking into account the increased likelihood of metastatic lymph nodes during surgical excision. It should not be overlooked that tumours might have multicentric characteristics in patients with low miR-125a levels, and the surgical resection area should be kept wide in suspicious cases.

Suppression of oncogenic miRNAs with miRNA inhibitors or supplementation of tumour suppressor miRNAs with synthetic miRNA mimics is being developed as a valuable experimental strategy for the treatment of cancer. Our data reveal that miRNAs are potential diagnostic and therapeutic targets in breast cancer.

## Data Availability

The datasets generated during and/or analysed during the current study are available from the corresponding author on a reasonable request.
